# Impact of *CYP2C19* Gene Variants on Long-Term Treatment with Atorvastatin in Patients with Acute Coronary Syndromes

**DOI:** 10.3390/ijms25105385

**Published:** 2024-05-15

**Authors:** Darius Čereškevičius, Vytautas Zabiela, Ali Aldujeli, Vaiva Lesauskaitė, Kristina Zubielienė, Vytautas Raškevičius, Ieva Čiapienė, Diana Žaliaduonytė, Agnė Giedraitienė, Vaidotas Žvikas, Valdas Jakštas, Vilius Skipskis, Olivija Dobilienė, Gintarė Šakalytė, Vacis Tatarūnas

**Affiliations:** 1 Institute of Cardiology, Lithuanian University of Health Sciences, Sukileliu 15, LT 50103 Kaunas, Lithuania; darius.cereskevicius@lsmu.lt (D.Č.); vytautas.zabiela@lsmu.lt (V.Z.); ali.aldujeli@lsmu.lt (A.A.); vaiva.lesauskaite@lsmu.lt (V.L.); vytautas.raskevicius@lsmu.lt (V.R.); ieva.ciapiene@lsmu.lt (I.Č.); vilius.skipskis@lsmu.lt (V.S.); gintare.sakalyte@lsmu.lt (G.Š.); 2 Department of Cardiology, Kaunas Hospital of the Lithuanian University of Health Sciences, Hipodromo 13, LT 45130 Kaunas, Lithuania; kristina.zubieliene@lsmu.lt (K.Z.); diana.zaliaduonyte@lsmu.lt (D.Ž.); 3 Institute of Microbiology and Virology, Lithuanian University of Health Sciences, Eivenių 4, LT 50161 Kaunas, Lithuania; agne.giedraitiene@lsmu.lt; 4 Institute of Pharmaceutical Technologies, Sukileliu 13, LT 50103 Kaunas, Lithuania; vaidotas.zvikas@lsmu.lt (V.Ž.); valdas.jakstas@lsmu.lt (V.J.); 5 Department of Cardiology, Lithuanian University of Health Sciences, Eivenių 2, LT 50009 Kaunas, Lithuania; olivija.dobiliene@lsmu.lt

**Keywords:** statins, atorvastatin, LDL cholesterol, CYP2C19, STEMI, NSTEMI

## Abstract

The effectiveness of lipid-lowering therapies may be insufficient in high-risk cardiovascular patients and depends on the genetic variability of drug-metabolizing enzymes. Customizing statin therapy, including treatment with atorvastatin, may improve clinical outcomes. Currently, there is a lack of guidelines allowing the prediction of the therapeutic efficacy of lipid-lowering statin therapy. This study aimed to determine the effects of clinically significant gene variants of *CYP2C19* on atorvastatin therapy in patients with acute coronary syndromes. In total, 92 patients with a confirmed diagnosis of ST-elevation myocardial infarction (STEMI) or non-ST-elevation myocardial infarction (NSTEMI) were sequenced for target regions within the *CYP2C19* gene on the Illumina Miniseq system. The CYP2C19 poor metabolizer phenotype (carriers of CYP2C19*2, CYP2C19*4, and CYP2C19*8 alleles) was detected in 29% of patients. These patients had significantly lower responses to treatment with atorvastatin than patients with the normal metabolizer phenotype. CYP2C19-metabolizing phenotype, patient age, and smoking increased the odds of undertreatment in patients (∆LDL-C (mmol/L) < 1). These results revealed that the CYP2C19 phenotype may significantly impact atorvastatin therapy personalization in patients requiring LDL lipid-lowering therapy.

## 1. Introduction

Hypercholesterolemia, or hyperlipidemia, is characterized by elevated levels of cholesterol in the blood and stands as a major risk factor for cardiovascular diseases, contributing significantly to the global burden of morbidity and mortality [1]. While lifestyle factors and dietary choices significantly impact cholesterol concentrations, genetic factors play a pivotal role in determining a predisposition to hypercholesterolemia [2]. A large study sponsored by the NIH (National Institutes of Health) discovered that a cholesterol-lowering diet and medical treatment decreased heart attacks in males with high cholesterol [3].

The discovery of compounds decreasing cholesterol levels in 1976 [4,5] has paved the way for a new class of drugs, 3-hydroxy-3-methyl-glutaryl-CoA reductase (HMG-CoA reductase, HMGCR) inhibitors or statins. *Goldstein* and *Brown* were the first to describe that patients with familial hypercholesterolemia who lack low-density lipoprotein receptors (LDLr) and produce increased amounts of cholesterol in response to the absence of these receptors [6]. It was assumed that inhibiting cholesterol through HMG-CoA reductase and the upregulation of LDLr is a key tool in the prevention of atherosclerotic cardiovascular disease [7,8]. In addition, statins can have a positive effect by stabilizing the atherosclerotic plaque and reducing atherothrombotic events through this mechanism [7].

The current European Society of Cardiology guidelines for the management of dyslipidemias [9] and American Heart Association guidelines for the Management of Patients With Chronic Coronary Disease [10] agree that primary prevention with statins, according to the patients’ risk category, should be initiated when lifestyle modification does not work and the low-density lipoprotein–cholesterol (LDL-C) goal is not achieved. For chronic coronary syndrome (CCS), familial hypercholesterolemia (FH), severe diabetes mellitus (DM), and chronic kidney disease (CKD) patients’ treatment should be initiated immediately after the diagnosis [10,11].

The main focus of the current guidelines is on lowering LDL-C through the use of drug combinations along with other lipid-lowering therapies [9,10]. Studies show that lipid-lowering therapies are insufficiently effective in patients, especially in high cardiovascular disease (CVD) risk patients [12,13,14,15,16]. Atorvastatin is among the most commonly prescribed statins. However, studies show it in adherence in patients with dyslipidemia [17].

In this era of precision medicine, better-customized statin therapy can be used. Some authors show that genetic variants can influence statin therapy. According to *Cano-Corres*, the *HMGCR* c.1564-106A>G variant reduces the effect of statins [18] *Maxwell* has examined a number of studies that have analyzed the influence of *ABCB1, APOE, KIF6, and TLR4* on the improvement of clinical outcomes during statin therapy [19]. The first gene-based clinical guideline on the use of statins approved by the Clinical Pharmacogenetics Implementation Consortium (CPIC) was published in 2012. The gene-based prescription of simvastatin was based on *SLCO1B1* variants. In 2014, the document was updated [20]. The latest “CPIC^®^ Guideline for Statins and SLCO1B1, ABCG2 and CYP2C9“ summarizes data from genotyping studies that demonstrate the influence of *SLCO1B1, ABCG2*, and *CYP2C9* on safer statin therapy that may allow the avoidance of statin-associated effects [21]. The guidelines described here aim to reduce the risk of statin-associated musculoskeletal symptoms (SAMS), as research studies show that statins can cause muscle pain. Still, this pain is rare and usually manifests as mild symptoms [22,23].

Some studies have shown that variants in *CYP2C19* may impact treatment effectiveness with statins [24]. Currently, no clear studies show that the effect of atorvastatin may depend on *CYP2C19* gene variants. It is worth noting that an individual carrying a non-functional allele such as CYP2C19*2, *3, or *4 will have impaired drug metabolism and is considered an intermediate metabolizer. In contrast, an individual carrying two non-functional alleles will be regarded as a poor metabolizer [25]. Currently, no guidelines allow for the prediction of the therapeutic efficacy of lipid-lowering statin therapy. Various studies show that linoleic acid derivatives may be significant in lipid metabolism [26]. Thus, this study aimed to determine the effects of *CYP2C19* gene variants and linoleic acid derivatives on atorvastatin therapy in patients with established cardiovascular disease.

## 2. Results

Among the patients, 30.4% were using statins before hospitalization, with 39.1% experiencing STEMI and 20.7% having undergone previous PCI (Table 1).

Of the patients with STEMI, 28.6% were on statins, and 43.8% were non-users *prior* to hospitalization (Table 2). No statistical significance was found between these two groups of patients.

Table 3 lists all variants detected in the *CYP2C19* gene. Of the variants that impact CYP2C19 functionality, rs4244285 was the most common. Two other variants, rs28399504 and rs41291556, described in the PharmVar database [27], were also detected, leading to reduced enzyme activity. One patient was found to be carrying a rare variant of uncertain significance.

Patients were divided into two groups based on their CYP2C19 metabolism: poor metabolizers and normal metabolizers. Carriers of the CYP2C19*2, CYP2C19*4, and CYP2C19*8 alleles were considered poor metabolizers, while all other patients were considered normal metabolizers. Normal metabolizers had a higher reduction in LDL-C (Table 4).

### 2.1. Regression Model

The multivariable logistic regression model showed (Table 5) that poor CYP2C19 metabolizing phenotype, patient age, and smoking increased the odds of undertreatment in patients (∆LDL-C (mmol/L) < 1) who received standard atorvastatin cholesterol-lowering therapy.

### 2.2. Analysis of Metabolite Data

**Figure 1:**
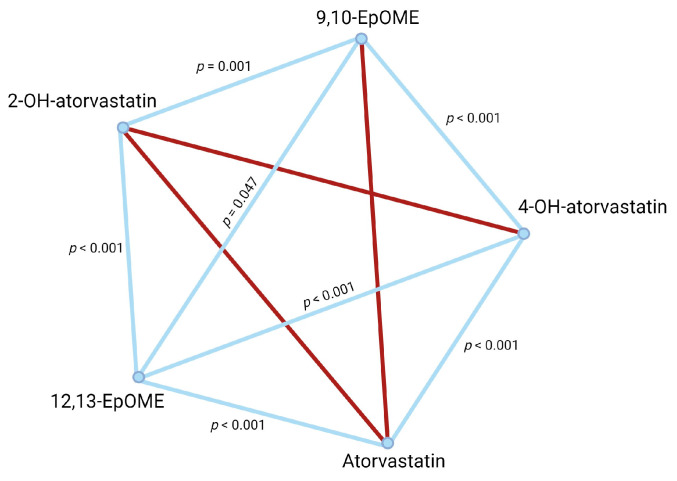
Pairwise analysis of compound concentrations in normal metabolizers. Significant associations are represented as blue lines. Non-significant associations are in red.

**Figure 2:**
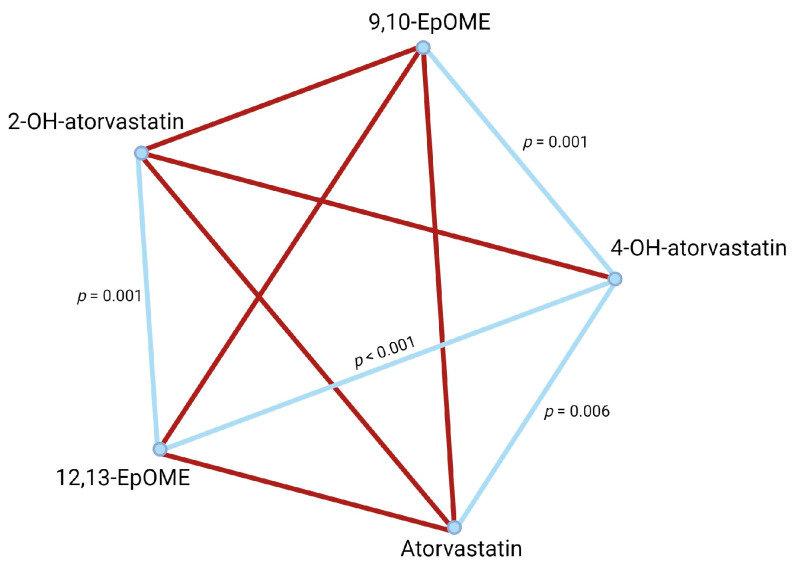
Pairwise analysis of compound concentrations in poor metabolizers. Significant associations are represented as blue lines. Non-significant associations are in red.

Pairwise analysis of plasma metabolite concentrations (Table 6) of atorvastatin, 4-OH-atorvastatin, 2-OH-atorvastatin, 9(10)-EpOME, and 12(13)-EpOME revealed that for normal metabolizers (Figure 1), 4-OH-atorvastatin concentration was dependent on atorvastatin concentration in blood plasma. Both 9,10-EpOME and 12,13-EpOME concentrations were associated with 4-OH-atorvastatin and 2-OH-atorvastatin plasma concentrations. There were different results in poor metabolizers (Figure 2): 4-OH-atorvastatin concentration was associated with atorvastatin and 9,10-EpOME and 12,13-EpOME concentrations. 12,13-EpOME concentration was associated with 2-OH-atorvastatin plasma concentrations.

## 3. Discussion

The results of this study have shown for the first time that *CYP2C19* variants may significantly affect atorvastatin lipid-lowering therapy. In addition, patient age and smoking were also associated with a decreased effect of atorvastatin therapy.

Statins are the cornerstone of lipid-lowering therapies for patients with cardiovascular disease. The European Society of Cardiology (ESC) guidelines divide patients into four risk categories: low, moderate, high, and very high risk. Lifestyle modification or drug therapy is considered according to the patient’s risk and LDL-C levels. If the primary (or secondary) prevention patient is without DM or FH but at very high risk, LDL-C reduction from baseline ≥ 50% and LDL-C goal of <1.4 mmol/L is recommended (I class, level C), and primary prevention for patients with high-risk LDL-C have a goal of <1.8 mmol/L (I class, level A). For individuals at moderate risk, the LDL-C goal is <2.6 mmol/L (IIa class, level A), and for low risk, <3 mmol/L (IIb class, level A). In most of the primary prevention cases, when lifestyle modification does not work and the desirable LDL-C-lowering effect is not achieved, statin therapy must be initiated [9]. Not only is the statin-induced LDL-C-lowering result relevant for secondary prevention patients, but its pleiotropic nature and their effect on CV morbidity and mortality are also vital for CVD prevention. The American Heart Association recommendations (2023) for chronic coronary syndrome (CCS) patients also support statin therapy to lower LDL-C to reduce the risk of major acute cardiovascular events (MACE) (class I, level A) [10].

Chronic oral statin treatment may have cardioprotective effects before STEMI [28]. Statins can prevent plaque rupture [29]. In a large study representing 5103 patients, 27% of the patients were low-intensity statin therapy users (LIST), and 17% used high-intensity statin therapy (HIST) before index acute coronary syndrome. In total, 56% were non-users (statin-naïve patients). The incidence of STEMI was lower in HIST patients than in LIST patients [30]. Our represented patients cannot confirm this effect because one of the limitations of this study was the relatively low patient number compared with the large-scale studies. However, the aim of this study was also different from the above-mentioned studies and was optimal for analyzing gene variants related to drug metabolism in the liver.

The most common loss-of-function variant in the represented patient group was rs4244285, also known as the *2 variant allele. *CYP2C19* loss-of-function alleles are known to affect the effectiveness of clopidogrel antiplatelet therapy, residual platelet aggregation, and a higher rate of stent thrombosis [31]. The natural function of CYP2C19 in the liver is the metabolism of various substrates. Studies have shown that the *CYP2C9* and *CYP2C19* variant alleles are associated with a higher prevalence of atherosclerosis in cigarette smokers [32]. In patients following PCI, *CYP2C19* loss-of-function alleles with peripheral endothelial dysfunction may predict future cardiovascular events [33]. Another study explained that a decline in epoxyeicosatrienoic acid concentrations due to *CYP2C19* variants may be associated with coronary microvascular dysfunction [34]. This same study also shows that increased epoxyeicosatrienoic acid concentrations lead to lower inflammation in the microvascular system. Inflammation has been shown to suppress CYP-450, including CYP3A4 [35]. Our data show that patients with normal CYP2C19 function had different atorvastatin metabolites and EpOMEs profiles compared to poor metabolizers. Researchers have shown that EpOMEs (vernolic acid (12,13-EpOME) and coronaric acid (9,10-EpOME)) are produced by activated neutrophils and macrophages during inflammation and are known as leukotoxins [36]. These compounds are linoleic acid derivatives produced by CYP450 enzymes [37]. EpOMEs are further metabolized into 9,10-dihydroxy-octadecamonoenoate (9,10-DiHOME) and 12,13-dihydroxy-octadecamonoenoate (12,13-DiHOME). The later compounds suppress neutrophil respiratory (or oxidative) bursts, impair immunological signaling [38], and probably impact the cholesterol metabolism or cholesterol-lowering effect of statins.

Rosuvastatin undergoes metabolism through CYP2C9 and CYP2C19; however, according to the literature, the main atorvastatin-metabolizing enzyme remains as CYP3A4 [39,40]. One study showed an effect of rosuvastatin but not atorvastatin on P2Y_12_ receptor reaction units (PRU) in patients with *CYP2C19* variant alleles and concomitant use of clopidogrel and statin [41]. A recent study examined the levels of LDL cholesterol in patients who were taking statins. The authors found that *CYP2C19* variants were associated with sdLDL-C levels and may predict the efficacy of statin therapy [24]. The results of our study revealed that reduction in LDL-C levels over six months was more effective in patients with normal metabolizer phenotypes of CYP2C19. Poor metabolizers had at least three times lower reduction in LDL-C blood plasma concentrations, despite the exact atorvastatin dosages as normal metabolizers (80 mg once daily). Our pioneering study describes the effect of the CYP2C19 phenotype on atorvastatin therapy in patients with acute coronary syndromes. Thus, the second limitation of this study is that functional studies were not performed to clarify the observations. However, one survey showed that CYP2C19 could metabolize atorvastatin lactone and 2-OH-atorvastatin lactone. The authors provide a more detailed description of how various statins can be metabolized. Atorvastatin is prescribed and used in the active acid form. Acid drugs are metabolized slower by liver cytochromes than more lipophilic lactone drugs. Under the action of certain enzymes, acid forms can be biotransformed into lactone forms and undergo metabolism through liver cytochromes [42]. Regarding the potentially inconsistent data on the impact of CYP2C19 on atorvastatin metabolism based on different studies, more detailed research is required to clarify the effect of atorvastatin metabolism in patients with acute coronary syndromes. The study utilized next-generation sequencing, a robust but time-consuming technique, to analyze variants of interest. However, alternative methods to sequencing can improve turnaround times while still detecting the most common variants in *CYP2C19* (*CYP2C19* *2, *4, *8 alleles) that have an impact. Real-time PCR or other alternative methods could be used for the fast detection of gene variants that may impact statin therapy, leading to precise cholesterol-reducing therapy with tailored treatments adopted for each patient individually.

## 4. Materials and Methods

### 4.1. Study Population and Inclusion Criteria

This study was a prospective, single-center investigation conducted at the cardiac intensive care unit of the Hospital of the Lithuanian University of Health Sciences Kaunas Clinics. The research included 92 consecutive patients admitted between January and November 2021, and all tested negative for COVID-19. These patients presented with either ST-Elevation Myocardial Infarction (STEMI) or Non-ST-Elevation Myocardial Infarction (NSTEMI). Each patient underwent invasive angiography followed by primary percutaneous coronary intervention (PCI). Patients were excluded from enrollment if they had a diagnosis of atrial fibrillation or pericardial diseases; a history of prior coronary artery bypass graft surgery; were pregnant; or had been diagnosed with significant structural heart diseases, including valvular heart diseases. Additionally, exclusion criteria encompassed patients with a documented history of hepatic, oncological, or lung diseases; allergy to contrast media; renal failure; and severe dementia.

Data collection included patient demographics, comorbidities, medications, and clinical course. STEMI, as per the 2023 ESC Guidelines, is marked by ST-segment elevation in two ECG leads, with specific thresholds for chest and limb leads and factoring in age and gender. A new left bundle branch block (LBBB) can also indicate STEMI. Diagnosis requires both ischemic symptoms and obstructive coronary artery disease confirmed by angiography. Without angiographic evidence of obstruction, a STEMI diagnosis is not made, even if ECG and clinical signs are present [43]. NSTEMI is identified through a 12-lead ECG showing a depressed ST-segment or T-wave inversion and a cardiac troponin rise exceeding five times the 99th percentile limit. Confirmation via coronary angiography is essential to diagnose the extent of arterial blockage [43]. All patients received standard treatment, including statins (80 mg of atorvastatin), angiotensin-converting enzyme inhibitors (or angiotensin receptor I blockers), β-adreno-blockers, and dual antiplatelet therapy (DAPT) comprising aspirin and a P2Y_12_ inhibitor such as ticagrelor or clopidogrel.

### 4.2. Lipid Profile Assessment

Lipid levels in our study were assessed upon admission and again at six months. For most lipids, enzymatic hydrolysis was used to measure total cholesterol, triglycerides, and HDL cholesterol, with colorimetric analysis indicating their concentrations. LDL cholesterol was estimated using the Friedewald formula (LDL cholesterol = total cholesterol − HDL cholesterol − (triglycerides/2.2)), effective when triglycerides are below 4.5 mmol/L. For higher triglyceride levels, direct LDL measurement was employed.

### 4.3. DNA Extraction and Sequencing

Genomic DNA extraction was performed employing standard laboratory procedures, and subsequent library preparation utilized Illumina DNA Prep kits (Illumina, San Diego, CA, USA) according to the manufacturer’s protocol. Specifically, a DNA quantity ranging from 200 to 500 ng underwent bead-linked tagmentation. After tagmentation, samples were subjected to amplification through 9 cycles of polymerase chain reaction (PCR). A unique pair of indices was assigned to each during the amplification process to ensure sample distinction. After amplification and a subsequent clean-up step, samples were equitably pooled, each comprising 12 samples.

A customized panel was employed for target region enrichment, utilizing xGen Lock Down probes from Integrated DNA Technologies (IDT, Coralville, IA, USA). This panel comprehensively covered all exons of *CYP2C19* included in the canonical transcript NM_000769.4, as per the National Center for Biotechnology Information (NCBI) Reference Sequence Database (https://www.ncbi.nlm.nih.gov/refseq, accessed on 1 October 2021). After enrichment, library pools underwent a sixteen-cycle PCR amplification. Subsequently, the prepared library pools were quantified using the Qubit High Sensitivity assay (Invitrogen, Carlsbad, CA, USA).

Sequencing was executed on an Illumina Miniseq system (Illumina, San Diego, CA, USA) utilizing the medium output 300 cycles kit. Data analysis was conducted with the Genomic Assembly Tool Kit (GATK) version 4.2.6.1, adhering to the best practice guidelines. This comprehensive methodology ensures robust and accurate interrogation of the genetic landscape, particularly focusing on the target region within the *CYP2C19* gene.

### 4.4. Metabolite Analysis: UPLC-ESI-MS/MS Conditions

Analysis of targeted compounds in human plasma samples was carried out with an Acquity H-class UPLC system (Waters, Milford, MA, USA) equipped with a triple quadrupole tandem mass spectrometer (Xevo TQD, Waters, Milford, MA, USA) with an electrospray ionization source (ESI) working in both positive and negative modes. An Acquity UPLC BEH C18 (100 × 2.1 mm 1.7 µm) column was used for the separation of the targeted compounds. The column temperature was maintained at 40 °C. Gradient elution was performed with a mobile phase consisting of 0.1% acetic acid water solution (solvent A) and acetonitrile (solvent B) with the flow rate set to 0.5 mL/min. Linear gradient profile was applied with the following proportions of solvent A: 0 to 1 min–75%, 8.0 to 8.5 min.–5%, 8.51 min; 75% total analysis time—10 min. Electrospray ionization was applied for analysis with the following settings: capillary voltage: 2.5 kV for negative mode and 3.5 kV for positive mode, source temperature: –120 °C, desolvation temperature: –400 °C, desolvation gas flow: 650 L/h, cone gas flow: 10 L/h. Collision energy and cone voltage were optimized for each compound separately (Table 7).

### 4.5. Statistical Analysis

Frequencies are presented in percentages. Quantitative clinical parameters were evaluated using a nonparametric Kruskal–Wallis test. Fisher’s exact test was used to assess the proportions of categorical variables. A *p*-value < 0.05 was considered statistically significant. All variables were chosen for the multivariable model by backward selection, with the final model containing only those with *p* < 0.05. According to Wang et al., 1 mmol/L reduction in LDL-C corresponds to a 19% lower risk of major vascular events and is independent of the baseline LDL cholesterol [44]. Therefore, a 1 mmol/L reduction in LDL cholesterol was used in this study to determine the relative efficacy of statin therapy.

Pairwise comparisons were used to evaluate the associations between atorvastatin, its metabolites (4-OH-atorvastatin, 2-OH-atorvastatin), and EPOME (9,10-epoxy-12Z-octadecenoic acid (9(10)-EpOME), as well as 12(13)epoxy-9Z-octadecenoic acid (12(13)-EpOME) concentrations in the represented patient sample.

## 5. Conclusions

The results revealed that the CYP2C19 phenotype may significantly impact atorvastatin therapy personalization in patients requiring LDL lipid-lowering therapy. However, more detailed studies are needed to show the exact role of CYP2C19 in determining the effect of atorvastatin.

## Figures and Tables

**Table 1:** Basic characteristics of the represented patient’s group.

Variable	*n*	%
Sex		
Men	49	53.3
Women	43	46.7
Smoking	54	58.7
STEMI	36	39.1
Hypertension	47	51.1
Diabetes mellitus	14	15.2
Renal insufficiency	11	12
Statins at hospitalization	28	30.4
Family anamnesis of ischemic heart disease	39	42.4
Previous PCI	19	20.7

**Table 2:** Patients with STEMI vs. NSTEMI according to statin therapy *prior* to hospitalization.

STEMI	On Statins	No Statin	Fisher’s Exact Test *p*
Yes *n* (%)	8 (28.6)	28 (43.8)	0.245
No *n* (%)	20 (71.4)	36 (56.3)	
Total *n* (%)	28 (100)	64 (100)	

**Table 3:** *CYP2C19* variants in the represented patient sample.

dbSNP rs ID	c.DNA Position (NM_000771.4)	AA Change (NP_000762.2)	Pharmvar Allele	Impact on Function	No of Het	No of Hom
rs3758581	c.991A>G	p.Ile331Val	-	-	7	0
rs17885098	c.99T>C	p.Pro33Pro	-	-	8	0
rs28399504	c.1A>G	p.M1V	CYP2C19*4	No function	2	0
rs58973490	c.449G>A	p.Arg150His	CYP2C19*11	Normal function	1	0
rs17878459	c.276G>C	p.Glu92Asp	-	-	12	0
rs17882744	c.1059C>T	p.His353=	-	-	1	0
rs3758580	c.990C>T	p.Val330Val	-	-	19	4
rs142974781	c.448C>T	p.Arg150Cys	-	-	1	0
rs41291556	c.358T>C	p.Trp120Arg	CYP2C19*8	No function	1	0
rs4244285	c.681G>A	p.Pro227Pro	CYP2C19*2	No function	19	4

**Table 4:** Patient CYP2C19 phenotype in the represented samples.

CYP2C19 Metabolizer Status	*n* (%)	LDL-C at Hospitalization (mmol/L) Median (Min-Max)	LDL-C 6 Months after Hospitalization (mmol/L) Median (Min-Max)	∆LDL-C (mmol/L) Median (Min-Max)	*p*-Value ***
Normal metabolizer	66 (71)	3.88 (2.87–6.36)	2.24 (1.17–5.41) **	1.69 (0.13–3.57)	0.006
Poor metabolizer *	27 (29)	3.78 (2.79–6.32)	2.78 (1.19–5.97) **	0.49 (0.01–4.09)	
Total	93 (100)	3.87 (2.79–6.36)	2.29 (1.17–5.97)		

* *CYP2C19* *2, *4, *8; ** the LDL-C values between two medians differed, *p* = 0.01; *** *p*-value between medians of ∆LDL-C.

**Table 5:** Variables that may decrease the effect of atorvastatin lipid-lowering therapy.

Variable	Odds Ratio	95% CI	*p*-Value
CYP2C19 poor metabolizer phenotype	7.027	(2.287–21.590)	0.001
Patient age in years	1.064	(1.017–1.113)	0.007
Smoking	3.396	(1.167–9.884)	0.025

**Table 6:** Atorvastatin, 4-OH-atorvastatin, 2-OH-atorvastatin, 9,10-EpOME, and 12,13-EpOME concentrations in the represented patient sample.

CYP2C19 Metabolizer Status	Atorvastatin in ng, Median (Min-Max)	4-OH-atorvastatin in ng, Median (Min-Max)	2-OH-atorvastatin in ng, Median (Min-Max)	9,10-EpOME, in ng, Median (Min-Max)	12,13-EpOME in ng, Median (Min-Max)
Normal metabolizer (*n* = 42)	6.5 (0–157.4)	0.5 (0–32.1)	4.1 (0–65.2)	21.3 (6.9–167.3)	37.4 (13–144.1)
Poor metabolizer (*n* = 21)	5.3 (0–60.1)	1.1 (0–8)	5.9 (0–24.8)	20 (0–148.7)	29 (0–180)
Total (*n* = 63)	5.3 (0–157.4)	0.6 (0–32.1)	4.5 (0–65.2)	20.5 (0–167.3)	34.8 (0–180)

**Table 7:** MS condition and MRM transitions for compounds of interest.

Compound	ESI Mode	Retention Time, min	Cone Voltage	Collision Energy	MRM Transition
Atorvastatin	Positive	5.68	30	40	559 > 250
Atorvastatin-d5 (IS)	Positive	5.68	30	40	564 > 255
2-Hydroxyatorvastatin	Positive	5.43	30	40	575 > 250
Parahydroxyatorvastatin, or Atorvastatin-4OH	Positive	4.42	30	40	575 > 250
20-HETE-d6 (IS)	Negative	6.45	50	12	325 > 307
9,10-Epoxy-12Z-octadecenoic acid (9(10)-EpOME)	Negative	6.79	30	20	295 > 171
12(13)Epoxy-9Z-octadecenoic acid (12(13)-EpOME)	Negative	6.74	30	20	295 > 195

## Data Availability

The datasets and resources generated during and/or analyzed during the current study are available from the corresponding author upon reasonable request.
